# Quantification and Detection of Ground Garlic Adulteration Using Fourier-Transform Near-Infrared Reflectance Spectra

**DOI:** 10.3390/foods12183377

**Published:** 2023-09-08

**Authors:** Michal Daszykowski, Michal Kula, Ivana Stanimirova

**Affiliations:** Institute of Chemistry, University of Silesia in Katowice, 9 Szkolna Street, 40-006 Katowice, Poland

**Keywords:** chemometrics, FT-NIR, fingerprinting, counterfeiting, classification, discrimination, Monte Carlo validation

## Abstract

This study demonstrates the rapid and cost-effective possibility of quantifying adulterant amounts (corn flour or corn starch) in ground and dried garlic samples. Prepared mixtures with different concentrations of selected adulterant were effectively characterized using Fourier-transform near-infrared reflectance spectra (FT-NIR), and multivariate calibration models were developed using two methods: principal component regression (PCR) and partial least squares regression (PLSR). They were constructed for optimally preprocessed FT-NIR spectra, and PLSR models generally performed better regarding model fit and predictions than PCR. The optimal PLSR model, built to estimate the amount of corn flour present in the ground and dried garlic samples, was constructed for the first derivative spectra obtained after Savitzky–Golay smoothing (fifteen sampling points and polynomial of the second degree). It demonstrated root mean squared errors for calibration and validation samples equal to 1.8841 and 1.8844 (i.e., 1.88% concerning the calibration range), respectively, and coefficients of determination equal to 0.9955 and 0.9858. The optimal PLSR model constructed for spectra after inverse scattering correction to assess the amount of corn starch had root mean squared errors for calibration and validation samples equal to 1.7679 and 1.7812 (i.e., 1.77% and 1.78% concerning the calibration range), respectively, and coefficients of determination equal to 0.9961 and 0.9873. It was also possible to discriminate samples adulterated with corn flour or corn starch using partial least squares discriminant analysis (PLS-DA). The optimal PLS-DA model had a very high correct classification rate (99.66%), sensitivity (99.96%), and specificity (99.36%), calculated for external validation samples. Uncertainties of these figures of merit, estimated using the Monte Carlo validation approach, were relatively small. One-class classification partial least squares models, developed to detect the adulterant type, presented very optimistic sensitivity for validation samples (above 99%) but low specificity (64% and 45.33% for models recognizing corn flour or corn starch adulterants, respectively). Through experimental investigation, chemometric data analysis, and modeling, we have verified that the FT-NIR technique exhibits the required sensitivity to quantify adulteration in dried ground garlic, whether it involves corn flour or corn starch.

## 1. Introduction

The adulteration of food and food products has been a well-known issue since ancient times [1]. Many products can be purchased in local markets thanks to the achievements of modern agriculture, improved cultivation, and effective distribution methods. In the past, access to different goods, particularly certain spices, was challenging and geographically limited. In addition to the availability and ease of product distribution, the price drives demand and supply. It remains the primary reason encouraging illegal attempts to counterfeit various food products.

The primary method of forgery, the so-called adulteration, consists in deliberately increasing the mass or volume through doping the product with a cheaper variant, and thus with much worse parameters, for example, caused by overlong or improper storage or adding not genuine parts of a plant. Sometimes, a completely different ingredient is introduced that acts as a filler. Another situation arises when carefully selected chemical components are added to fortify the overall flavor and improve the perception of a sample. For instance, black pepper is tampered with piperine, chili pepper with curcumin, and ginger with capsaicin. When color reflects quality, non-permitted dyes such as carcinogenic Sudan I–IV, metanil yellow, dimethyl yellow, and rhodamine B dyes are used. All of these illegal adulteration practices allow for the inflation of the price of a product.

Unfortunately, basic spices and their mixtures are frequently adulterated [2]. The doping procedure is easy to hide when the final product has powdered form or is highly processed. The expected level of adulteration and its effectiveness depends on the characteristics of the main product, adulterant(s), consumer experience, and economic factors, including the product’s price, consumer demand, and scale of production. Every day, the consumer can use only his/her sense organs to evaluate primary quality attributes such as the smell, structure, and color of a commodity. The added substances intentionally have a similar particle size, color, and smell, so their presence can fool consumers’ senses. For this reason, detecting food adulteration requires sophisticated and sensitive instrumental techniques combined with chemometric data processing, offering objective judgment.

In the literature, many adulteration attempts have been described [3]. The most shocking public opinion cases involve adultery of spices or food products with toxic substances such as metal salts and non-permitted dyes. For instance, in Hungary in the 1990s, the Hungarian Ministry of Agriculture found out that approximately 5.8% of 3432 randomly sampled powdered red pepper samples contained very toxic lead (III) oxide [4]. The ingestion of the toxic spice caused the deaths of several people, and many were severely poisoned with lead. Moreover, it resulted in the collapse of Hungarian paprika exports [1].

Considering the chemical complexity of spices and aromatic herbs and their form, detecting adulterants and their reliable quantification are very challenging. Various analytical techniques, including spectroscopy and chromatography, have been proposed to fulfill this goal [5,6,7]. The most appreciated methods in the analysis of diverse chemical food components are flame atomic absorption spectroscopy, graphite furnace atomic absorption spectroscopy, inductively coupled plasma optical emission spectroscopy, and energy-dispersive X-ray fluorescence. They are well-suited for elemental analysis and the determination of heavy metals that may threaten consumers’ health and life. On the other hand, tracing the presence of organic adulterants may require separating mixture components. For this purpose, high-performance liquid and gas chromatography are the most popular methods. They can be combined with different single- (e.g., UV, fluorescence, flame ionization) and multi-channel detectors (e.g., diode-array detector, mass spectrometry), enabling the analysis of a wide range of compounds. Since they have the potential to separate and quantify individual components of a complex mixture, detecting adulterants becomes easier. However, before analysis, samples often require extensive preparation. To obtain a satisfactory resolution, the optimization of separation conditions is necessary. Moreover, quantification and identification require expensive standards, but they are not always available. Therefore, chromatographic and spectroscopic fingerprinting strategies combined with the chemometric modeling of spectroscopic fingerprints are attractive alternatives, for instance, UV-Vis, infrared, near-infrared (NIR), and Raman spectroscopy [5,7,8,9]. NIR spectroscopy has several advantages that considerably extend the use of this technique in many fields of analysis, including applications specifically focused on determining the authenticity of various products and spices [10,11]. NIR spectroscopy is mainly valued for little or no sample preparation, the possibility to analyze samples in different forms, rapid and cost-effective analysis, remote measurements, and ongoing miniaturization. It has also demonstrated great potential in detecting adulteration and the authentication of diverse food commodities (e.g., [12,13,14,15,16]).

Surprisingly, counterfeiting is not limited solely to expensive spices, such as saffron. Even basic and relatively cheap ones are tempting targets for fraudsters thanks to a large scale of turnover and the possibility of product repackaging before entering local markets [2]. Garlic granulate is imported to Europe mainly from China, which is the most significant worldwide producer. According to Tridge (https://www.tridge.com/, accessed on 28 June 2023), global sourcing hub of food and agriculture, in 2018, garlic production in China reached 21.2 M tonnes. Regarding the production scale and high consumer demand, garlic is one of the primary candidates for adulteration. As explained in reference [7], other factors may also considerably inflate the price of garlic, making it more prone to falsification, for instance, poor weather conditions.

Numerous substances are recognized as potential adulterants of dried ground garlic, including corn starch, corn flour, potato starch, potato flour, rice flour, talc, chalk, maltodextrin, cassava, and white corn meal. Unfortunately, this list remains incomplete. Our study verified the ability to distinguish two adulterants, corn flour and corn starch, both originating from the same plant material. Their choice for our research is supported by the following facts. Corn flour and corn starch are easily accessible materials on a global scale. They can be procured in significant quantities without arousing suspicion. Their consumption does not pose health risks. These two adulterants share a similar resemblance in both appearance and texture to ground and dried garlic. They have a neutral flavor and are odorless. Furthermore, the particle size, bulk, and volume of the mixed materials are also comparable. Consequently, blending dried ground garlic with corn flour or corn starch in varying proportions is straightforward, yielding adulterated products with similar density and packaging volume. All of these attributes firmly position flour and starch high on the list of possible adulterants for ground garlic. However, the efficient and cost-effective detection and quantification of corn flour or corn starch present considerable analytical challenges. The main goal of this study is to demonstrate the usefulness of the FT-NIR technique combined with chemometric data modeling as a rapid method for quantifying these adulterants.

## 2. Materials and Methods

### 2.1. Samples

All substances used in the experiment were purchased from a local Polish importer: garlic powder (0.5 kg, country of origin: China), corn starch (1 kg, country of origin: Serbia), and corn flour (1 kg, country of origin: Poland). Materials were stored in original packaging with a zip closure and in an additional package to protect them against the influence of external factors.

Binary mixtures containing garlic powder and one adulterant (corn starch or corn flour) were weighted using an analytical balance (Radwag XA 100/2X, Poland) with 0.1 mg precision. The material (2.00 g) was prepared directly in glass vials with screw caps and tightly closed, which allowed for subsequent storage of samples. Samples represented 25 concentration levels with the following weight percentages of adulterant: 0%, 0.5%, 1%, 2%, 2.5%, 3%, 4%, 5%, 7.5%, 9.5%, 10%, 11.5%, 13.5%, 15%, 17.5%, 20%, 22.5%, 25%, 27.5%, 30%, 32.5%, 35%, 50%, 60%, 70%, and 100%. They were prepared and weighed in random order.

### 2.2. Measurement of the Fourier-Transform Near-Infrared Spectra

The Fourier-transform near-infrared spectra (FT-NIR) were measured using the Antaris II FT-NIR analyzer (Thermo Scientific, Waltham, MA, USA). The instrument was equipped with an integrating sphere enabling diffuse reflection analysis of different materials, including powdered samples. The FT-NIR absorbance spectra were collected between 10,000 cm^−1^ and 4000 cm^−1^ with 2 cm^−1^ resolution, i.e., between 1000 nm and 2500 nm, directly through glass vials. Before collecting the spectra of adulterated ground garlic mixtures, moisture was removed from the samples through drying them to constant weight in a laboratory dryer at 80 °C. Then, they were carefully mixed to obtain an even distribution of an adulterant in a sample. Finally, each mixture was described using the average FT-NIR spectrum of 32 independent scans. Spectroscopic measurements were carried out at stable room temperature (22 °C) and humidity (50%). Spectra were recorded thirty minutes after switching on the instrument in order to stabilize the radiation source. The background spectrum was recorded at the beginning of the measurements. The background correction procedure was repeated automatically every hour using an internal standard (diffusely reflective gold plate).

### 2.3. Exploratory Analysis of the FT-NIR Spectra

Principal component analysis (PCA) is a projection technique that projects multivariate data onto a space spanned by a few principal components [17]. Their construction assumes preserving a maximal amount of data variance explained simultaneously by the smallest number of latent variables, called principal components (PCs). Principal components are new orthogonal variables summarizing the structure of multivariate data. They are linear combinations of explanatory variables. This is the most helpful advantage of the PCA model, opening the possibility for efficient data compression and exploration. PCA models data variance as a product of a few first scores and loadings, containing projections of samples and explanatory variables onto principal components. Information carried by scores and loadings assists in visually assessing data structure, particularly when studying similarities observed among samples and variables.

### 2.4. Construction of Multivariate Calibration Models

Constructing calibration models requires finding a mathematical relationship between a dependent variable and one or more explanatory variables. It generally helps to replace reference measurements with a more straightforward approach. Near-infrared spectroscopy is an excellent example of the so-called fingerprinting technique, which is fast, non-destructive, cost-effective, and able to characterize complex samples according to unique spectra containing chemically relevant information. However, many bands overlap in the NIR range, and selecting one spectral feature relevant from the calibration perspective is impossible. Then, for calibration, more informative spectral features are needed. However, if the number of explanatory variables exceeds the number of samples, many variables are mutually correlated, and the least squares method cannot be used to determine the regression coefficients.

Principal component regression (PCR) and partial least squares regression (PLSR) are two well-suited methods for modeling highly correlated explanatory variables. They establish the relationship between the dependent variable (a response) and a few orthogonal latent variables. The fundamental difference between PCR and PLSR models arises from how latent variables are constructed [18]. In PCR, spectral variables are replaced with a few first orthogonal scores obtained from PCA. They summarize the variance of explanatory variables and the structure of multivariate data. In PLSR, latent variables are constructed to explain the maximal variance of explanatory variables and the maximal covariance between latent variables and the modeled response. The model is built for a set of representative calibration samples, and the number of latent variables is optimized to minimize the risk of over-optimistic predictions. The model’s performance is usually evaluated using such figures of merit as the mean squared error of prediction and coefficients of determination calculated for calibration and validation samples. A satisfactory model offers predictions characterized by a relatively low mean squared error of prediction for calibration and test samples (RMSEC and RMSEP), with a slight difference between these two figures. At the same time, the coefficients of determination are expected to be close to unity. In this study, the optimal model complexity was determined using leave-one-out cross-validation and examining the prediction errors for calibration and validation samples.

When the relationship is linear, and there are no outlying samples in calibration data, further model improvement is possible if undesired sources of variability (e.g., baseline and scattering) are effectively removed from the spectra. The negative influence of these two signal components can be suppressed through transforming the spectra with various preprocessing techniques [19]. The most popular ones are normalization to the unit variance, derivatives, detrending, standard normal variate (SNV), multiplicative scatter correction (MSC), extended multiplicative scatter correction (EMSC), inverse scattering correction (ISC), and extended inverse scattering correction (EISC). Sometimes it is necessary to apply more than one but in careful order. Unfortunately, there are no strict guidelines regarding the selection of preprocessing methods. Therefore, many are extensively tested while constructing multiple calibration models. The final decision regarding optimal spectral preprocessing results from analyzing the prediction power of a calibration model.

### 2.5. Discrimination and Classification Models

Partial least squares discriminant analysis (PLS-DA) is a variant of PLSR where the modeled response includes information about to which group each sample belongs to [20]. Its simplest variant discriminates two groups of samples, with ‘−1’ and ‘+1’ labels, in the space of a few latent variables. The model is built for representative training samples, describing diverse sources of variability. Based on spectra, the optimal PLS-DA model provides the so-called ‘hard’ logic rule assigning training, validation, and any new sample to one of the two groups.

OC-PLS is a classification approach that constructs ‘soft’ logic rules for each group of samples independently [21]. Similarly to PLS-DA, the training of a model requires a representative set of samples. Soft logic rules are derived based on Mahalanobis distances calculated in the space of latent variables and model residuals. The OC-PLS models classify training and validation samples to one group, more than one group, or any, depending on whether or not the Mahalanobis distances and model residuals calculated for the tested samples exceed the corresponding threshold values. Contrary to ‘hard’ classification, models involving the formulation of ‘soft’ logic rules are suitable when the number of groups is not known a priori, as is the situation when the studied samples could be adulterated with various adulterants.

The performance of discriminant and classification models is usually scored according to the correct classification (or discrimination) rate (accuracy), sensitivity, and specificity. These figures of merit are based on the number of observed true positives (TPs) and true negatives (TNs) and the corresponding model predictions: false positives (FPs) and false negatives (FNs). Accuracy is defined as the ratio of correctly recognized samples to all samples. Sensitivity is the ratio between TPs and the sum of TPs and FNs. Specificity is the ratio between TNs and the sum of TNs and FPs.

## 3. Results

### 3.1. The FT-NIR Spectra of Pure Components

**Figure 1:**
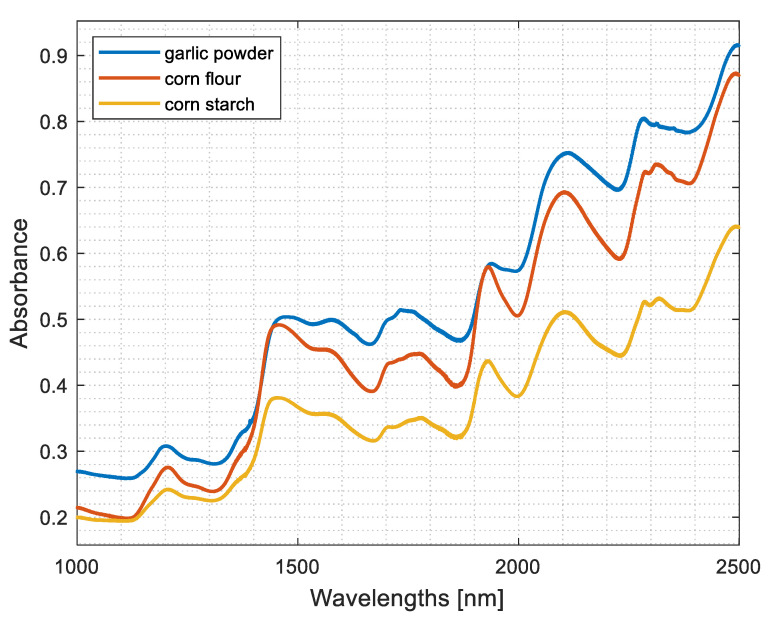
Fourier-transform near-infrared spectra of pure components: ground garlic, corn flour, and corn starch.

Figure 1 presents three FT-NIR absorbance spectra of dried samples of pure compounds: ground garlic, corn starch, and corn flour. Their spectra are very similar because as plant-derived material, ground garlic, corn starch, and corn flour differ in the amounts of proteins, fiber, minerals, and vitamins. In addition, in ground garlic, there are sulfur-containing compounds (allicin, alliin, diallyl sulfide, diallyl disulfide, and diallyl trisulfide), flavonoids, alliinase, amino acids (including cysteine and methionine), polyphenols, water-soluble compounds, and lipids in small amounts. The FT-NIR spectra of garlic have characteristic absorption bands that can be found at 1100–1225 nm (II overtone C–H stretching), 1400–1500 nm (I overtone N–H stretching, and I overtone O–H stretching), 1650–1800 nm (I overtone C–H stretching and I overtone S–H), 1800–2000 nm (II overtone carbonyl group), 2000–2200 nm (combination N–H stretching and combination O–H stretching), and 2200–2450 nm (combination C–H stretching) [22].

Corn starch is extracted from the internal part of the corn kernel, and most of the proteins, fiber, vitamins, and minerals are removed during production. Therefore, it contains mainly carbohydrates from starch molecules. On the other hand, corn flour is obtained through grinding whole corn kernels and thus has more proteins, fiber, minerals (mainly iron, potassium, magnesium), and B vitamins. The typical absorption bands in the corresponding FT-NIR spectra arise mostly from starches and cellulose and are associated with fiber as cellulosics and as lignins. A detailed list of the corresponding absorption bands can be found in reference [23].

It becomes evident that the spectrum of corn starch is the most dissimilar compared to the spectra of garlic powder and corn flour. These differences are expressed as a systematic increase in absorbance values in the upper range of the NIR spectrum, namely from 1400 nm. Differences between these spectra can be found around 1445 nm, 1930 nm, and 2225 nm.

### 3.2. Exploratory Analysis of FT-NIR of Adulterated Samples

**Figure 2:**
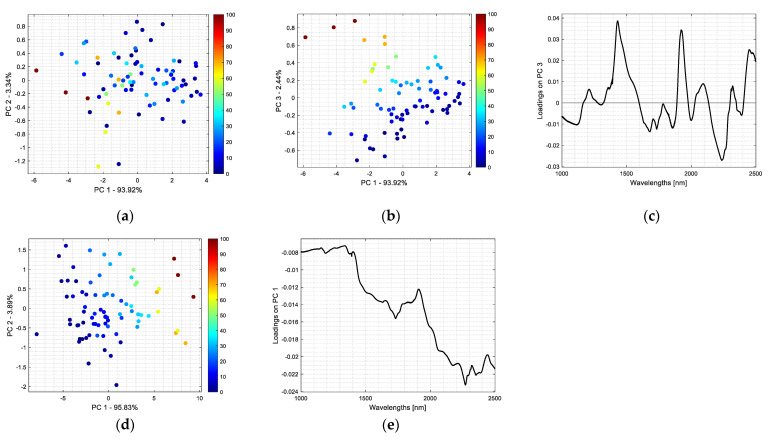
Projection of the FT-NIR spectra of ground and dried garlic samples adulterated with corn flour onto (**a**) PC 1 and PC 2 as well as (**b**) PC 1 and PC 3. (**c**) Loading values on PC 3. Projection of the FT-NIR spectra of ground and dried garlic samples adulterated with corn starch onto (**d**) PC 1 and PC 2. (**e**) Loading values on PC 1.

Figure 2a,b display two projections of the FT-NIR spectra that characterize ground garlic samples adulterated with corn flour. Their spatial distributions are shown on planes spanned by selected pairs of principal components (scores projections). They describe a large portion of the total data variance in the amount of 97.26% (see Figure 2a) and 93.36% (see Figure 2b).

Every dot on a score projection has a color reflecting adulterant concentration, expressed as a percentage of the total sample weight, and a color bar indicates the concentration gradient. Figure 2c displays loading values on the third principal component as a function of wavelengths.

Figure 2d presents a projection of samples on the first two principal components. The PC 1–PC 2 plane uncovers 99.72% of the total spectral variability. In Figure 2e, the loading values for the first principal component are plotted versus corresponding wavelengths.

### 3.3. Multivariate Calibration Models Based on Latent Variables

The performance of all latent variable models constructed in this study is visualized as a diagram where predicted values of adulterant concentration using a model are plotted versus the observed concentration. In these diagrams, black lines (with a slope equal to one) represent an ideal situation, i.e., no difference between observed values of the dependent variable and the predicted ones using a given calibration model. Calibration and independent validation samples are indicated as dots and circles, respectively.

Moreover, 56 different preprocessing methods and their sound combinations were exhaustively tested for each calibration model. These included signal normalization to the unit variance, derivatives (the first and second derivatives, including Savitzky–Golay signal smoothing), detrending, standard normal variate (SNV), multiplicative scatter correction (MSC), extended multiplicative scatter correction (EMSC), inverse scattering correction (ISC), and extended inverse scattering correction (EISC). In our study, 1680 calibration models were examined to find the optimal preprocessing scheme (56 × 15 = 840 for each type of modeling method). The optimal spectral preprocessing scheme led to models with the smallest possible number of latent variables and the smallest and the most comparable root mean squared errors obtained for calibration and validation samples.

Calibration models were developed using the exact calibration and validation sets. Calibration samples were selected to cover entire calibration range. In total, fifteen samples with different concentration levels of a single adulterant were considered: 0%, 0.5%, 1%, 2%, 3%, 5%, 10%, 15%, 20%, 25%, 30%, 35%, 50%, 70%, and 100%. All samples were prepared in triplicate. Thus, the calibration set contained 45 samples. In the validation set, there were 33 samples representing eleven concentration levels: 2.5%, 4%, 7.5%, 9.5%, 11.5%, 13.5%, 17.5%, 22.5%, 27.5%, 32.5%, and 60%.

**Figure 3:**
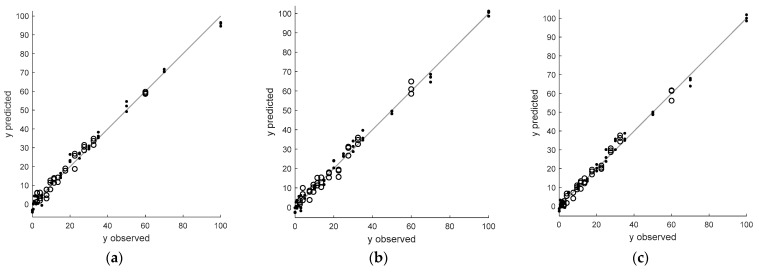
Principal component regression models constructed for (**a**) original FT-NIR spectra of ground and dried garlic samples adulterated with different amounts of corn flour, (**b**) original FT-NIR spectra of ground and dried garlic samples adulterated with different amounts of corn starch, and (**c**) FT-NIR spectra of ground and dried garlic samples adulterated with different amounts of corn starch after the optimal transformation (standard normal variate).

Figure 3a presents the principal component regression model. It was constructed for the original FT-NIR spectra describing ground and dried garlic samples adulterated with different amounts of corn flour. Figure 3b,c displays the predictions of two PCR calibration models constructed for the original spectra and the optimally transformed ones estimating the amount of corn starch added to ground garlic samples.

**Figure 4:**
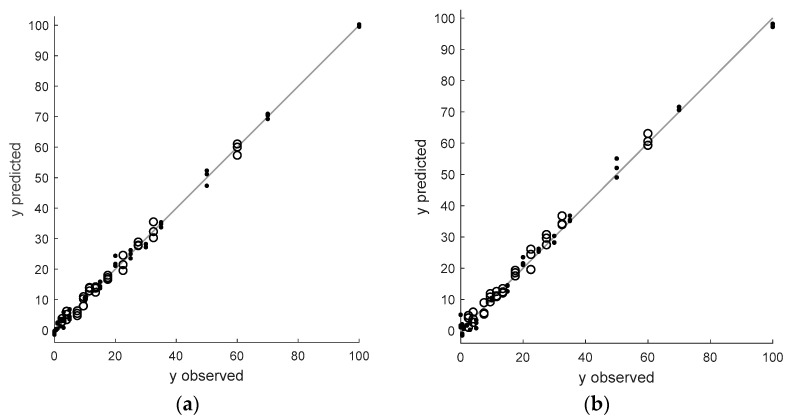

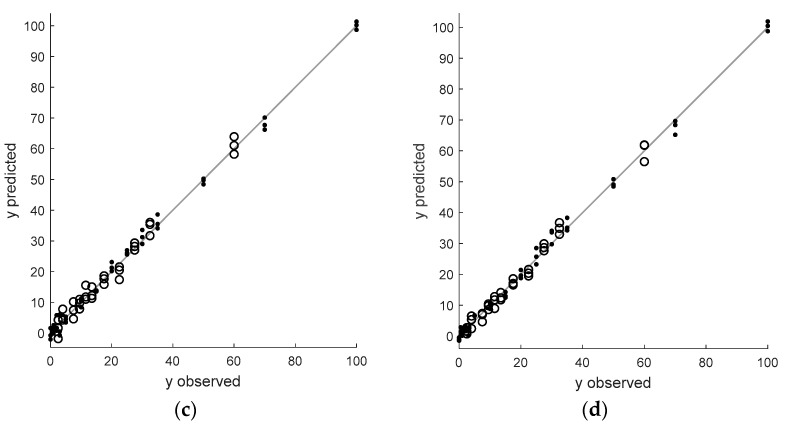
Partial least squares regression models for (**a**) original and (**b**) FT-NIR spectra of ground and dried garlic samples adulterated with different amounts of corn flour after optimal transformation (standard normal variate); (**c**) original and (**d**) FT-NIR spectra of ground and dried garlic samples adulterated with different amounts of corn starch after optimal transformation (the first derivative, window equal to fifteen sampling points and polynomial degree equal to two).

The predictions obtained from the partial least squares regression models, built for original and optimally preprocessed spectra for ground and dried garlic samples adulterated with corn flour or corn starch, are shown in Figure 4.

Table 1 presents an overview of a few selected figures of merit obtained from the latent variable models built in this study. It includes information about the optimal pretreatment of the FT-NIR spectra and the optimal number of latent factors, *f*, (model complexity). Each calibration model was characterized by the root mean squared error calculated for calibration (RMSEC) and validation samples (RMSEP) as well as the coefficients of determination for calibration (R^2^c) and validation samples (R^2^v).

Regardless of the model type and the considered adulterant, the variances of predictions for replicate samples from the calibration set, representing different concentration levels, are similar. This fact indicates that there is no proportional error, and the models offer the same predictive power across the maximal calibration range (0-100% *w*/*w* of adulterant).

### 3.4. Discrimination of Adulterated Samples Using PLS-DA

The PLS-DA model was constructed to discriminate samples adulterated with corn flour or corn starch, denoted as −1 and +1, respectively. Table 2 summarizes the correct classification rates, sensitivities, and specificities of the optimal model with seven latent factors calculated for samples from the training, test, and validation sets. All figures of merit were reported with their uncertainties, expressed as the corresponding standard deviations. Uncertainties associated with different figures of merit were estimated using the Monte Carlo approach and reported as the average of 500 iterations [24]. Before model construction, available samples were split into model and validation sets, as in the case of the calibration step. The model set included 50 samples from each group, and the validation set included 25. At each Monte Carlo iteration, 35 samples were selected randomly from each group of the model set, and they were included in the training set. The remaining 15 samples formed an internal test set.

**Figure 5:**
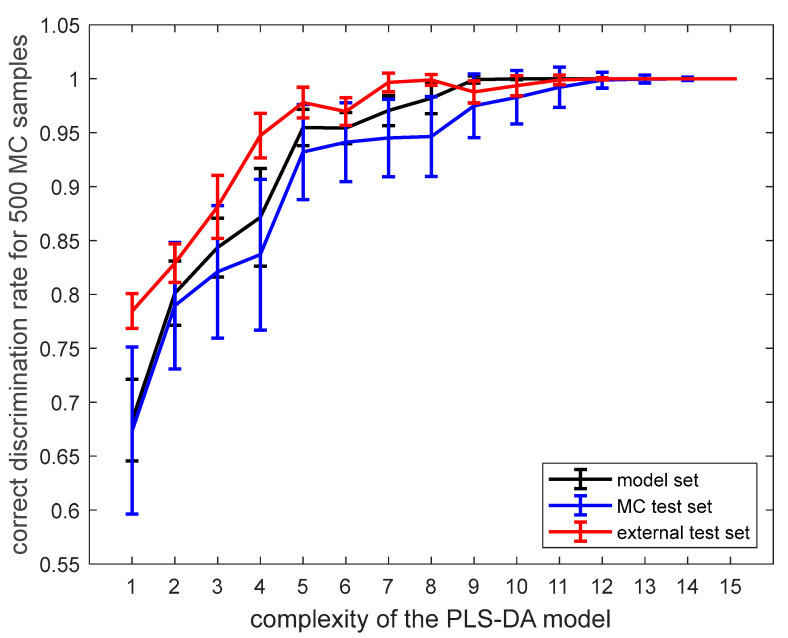
Average correct discrimination rates obtained for the model set, test set, and external test set from partial least squares discriminant model as a function of model complexity with indicated uncertainties estimated using the Monte Carlo validation approach (500 random subsamples).

In Figure 5, the average correct classification rates are presented as a function of model complexity along with their uncertainties of estimation. The optimal PLS-DA model included seven latent variables, and their number was selected based on evaluating the correct discrimination/classification rates.

### 3.5. Classification Models

Table 3 summarizes results obtained from the OC-PLS models constructed for garlic adulterated with corn flour and corn starch. They are characterized by sensitivity, specificity, and the correct classification rate. The optimal complexity of the classification models was determined using the cross-validation procedure. Both OC-PLS models included seven latent factors.

## 4. Discussion

### 4.1. Exploratory Analysis

Analysis of the information revealed in the projection of scores, shown in Figure 2a, allows us to conclude that the first two principal components account for 93.92% and 3.34% of the spectral variability. Even though the amount of modeled variance is large, it does not explain changes in the concentration of corn flour. Only the third principal component captures this expected trend, which explains a tiny portion of the total data variance (2.44%). Samples of pure ground garlic have the lowest negative score values on the third principal component. With an increasing adulterant concentration in the samples, their score values become larger. For spectra of pure corn flour, they reach positive values above 0.6 (see Figure 2b). The analysis of loading values on the third principal component highlights four relevant spectral bands (around 1430, 1924, 2235, and 2460 nm) that enhance the interpretation of adulterant concentration in terms of absorption bands. The corresponding loading values of the third principal components have large absolute values (see Figure 2c). Pure ground garlic samples and samples containing low concentrations of corn flour have negative score values on PC 3 and large negative values of loadings around 2235 nm. Therefore, they absorb more in this spectral region compared to the respective absorption regions of FT-NIR spectra, characterizing highly adulterated samples. As indicated in Figure 2c, loadings corresponding to spectral bands found around 1430, 1924, and 2460 nm are positive. This means that the absorbance intensity for these bands is relatively low. On the other hand, absorption behavior is the opposite in the four discussed spectral regions for samples with larger concentrations of added corn flour.

As indicated in Figure 1, the FT-NIR spectra of corn starch indicate much smaller absorption in the upper spectral range compared to the spectra of pure ground garlic and the spectra of pure corn flour samples. Therefore, the first principal component reveals changes in adulterant concentration very well. Negative score values on the first principal component have samples with concentrations of corn starch up to 30%, whereas pure ground garlic samples have the lowest score values (see Figure 2d). All loading values on the first principal component are negative. It points out a systematic difference in the spectra: low absorption in the entire spectral range. Moreover, increasing adulterant concentration decreases absorbance. This effect becomes more prominent beginning at approximately 1900 nm (see Figure 2e).

### 4.2. Construction of Multivariate Calibration Models

In this study, two types of calibration models were constructed-PCR and PLSR. Regardless of the calibration problem, all models indicate a very good linear relationship between spectra (represented by latent variables) and the response. A more detailed discussion of each calibration problem is provided below.

The initial PCR model was built to estimate concentrations of corn flour based on original FT-NIR spectra. Only three latent variables were necessary to obtain relatively good predictions for calibration and validation samples. The predictions presented in Figure 3a and their relatively small residuals confirm a very good linear fit of the PCR model to calibration and validation data, regardless of the concentration level of the adulterant. Moreover, the model has comparable prediction errors for calibration and validation samples. They are below 2.53%, considering the total calibration range (see Table 1). Its RMSEC and RMSEP values are equal to 2.5397 and 2.3115, respectively. Moreover, the corresponding coefficients of determination are both very high, above 0.97. This means that spectral data contain relevant information that can explain ca. 97% of the response variance. The further improvement of the calibration model requires removing undesired sources of variance from the FT-NIR spectra. However, for this type of adulterant, evaluating the performance of PCR models built for 56 sets with FT-NIR spectra that were preprocessed differently indicated that the optimal model was achieved using the original spectra.

When the concentration of corn starch in ground garlic samples is modeled, the PCR model for the original spectra is more complex than the previous one. It requires nine latent variables and offers root mean squared errors for calibration and validation samples equal to 2.3140 and 2.6896, respectively (see Table 1 and Figure 3b). These values correspond to a prediction uncertainty of less than 2.69% of the calibration range. Both coefficients of determination exceed 0.97. After selecting the optimal spectral preprocessing method (standard normal variate), the final complexity of the PCR model decreased from nine to four latent variables. In addition to this benefit, removing part of the scattering effect from FT-NIR spectra slightly decreased the RMSEC and RMSEP values (see Table 1 and Figure 3c).

In most applications, PCR and PLSR have very similar performance, but in this study, the PLSR models provide better predictions for calibration and validation samples regardless of the considered adulterant type. The predictions of the calibration model with six latent factors, constructed to describe the concentration of corn flour in the test samples, were lower than 1.56% considering the calibration range (see Table 1 and Figure 4a). After transforming the FT-NIR spectra using the first derivative, the model complexity decreased to four factors, but its prediction error slightly increased (see Table 1 and Figure 4b). The optimal PLSR model for the original spectra of samples adulterated with corn starch, with eight latent variables, offers predictions for calibration and validation samples with root mean squared errors smaller than 2.23% regarding the entire calibration range (see Table 1). After transforming the NIR spectra with the inverse scatter correction (ISC) method, these errors decreased and were eventually lower than 1.78. Residuals from the calibration model shown in Figure 4d appear to follow the normal distribution more than that of the model built for the original spectra (see Figure 4c).

### 4.3. Discrimination of Samples with Two Adulterants Using PLS-DA

The results provided in Table 2 demonstrate that it is possible to discriminate between two groups of samples regarding the type of adulterant. Their separation is carried out in the space of latent variables constructed using the PLS-DA approach. In other words, FT-NIR spectra capture relevant chemical differences, helping separate the two groups of samples. The optimal discriminant model with seven latent variables performs very well. It offers very high accuracy. For the independent test set, it is equal to 99.66%. Sensitivities and specificities for training, test, and validation samples are also very high (above 99% for the test set). Moreover, all of these figures of merit have relatively small uncertainties, as demonstrated in Table 2 and Figure 5.

### 4.4. Classification OC-PLS Models

The PLS-DA model discriminates between groups of samples using so-called ‘hard’ classification logic rules. This approach is recommended to explore or verify existing differences between groups of samples. Hard classification rules enable the assignment of samples based on the model parameters and their physicochemical characteristics to one group represented by the samples from a training set. Since many possible adulterants exist, some of which are unknown, detecting adulterant types requires considering so-called ‘soft’ classification rules. This strategy assumes constructing logic rules for each group of samples and then verifying whether a sample belongs to a given group. As a result of this evaluation, a sample is assigned to one group or more groups, or it is labeled as unclassified. Such a classification philosophy is appropriate when the number of possible groups is unknown a priori. In this study, OC-PLS models were constructed to verify whether detecting a particular adulterant type (corn flour or corn starch) is possible. The obtained figures of merit for both classification models indicate that they can adequately recognize samples from the same group (see Table 3). In other words, the number of true positive samples is very high, leading to the very high sensitivity of the classification model. Unfortunately, both classification models tend to accept too many samples from another group, increasing the number of false positives, which decreases the specificity of the classification model. Regarding the classification model constructed to recognize garlic samples doped with corn flour, its sensitivity exceeds 97%. When the OC-PLS model is validated using samples adulterated with corn starch, its specificity reaches only 64%. The classification model built to identify corn starch in samples has even worse specificity (45%) than the previous model.

## 5. Conclusions

Near-infrared spectra can be used to develop sound calibration models to predict the amount of adulterant in ground and dried garlic. In general, PLSR models performed better than PCR models regarding prediction errors and the coefficient of determination for calibration and validation sets. The further improvement of the calibration models was possible via including spectral preprocessing. The exhaustive testing of how 52 different preprocessing schemes influence the prediction of the calibration models enabled the selection of the optimal preprocessing approach.

Even though spectral differences between corn flour and corn starch are relatively small, it was possible to discriminate between the two groups of adulterants using the PLS-DA approach. The discriminant model was characterized by very high correct classification rates for the validation samples (above 99%). However, results obtained from OC-PLS indicate that the original FT-NIR spectra do not contain sufficient selective information to derive classification models with high specificity for these two types of adulterants.

With the rapid development of miniaturized NIR instruments and their wider implementation, the detection of adulteration will likely become cheaper, faster, and easier. Through enhancing chemometric models, optimizing spectral preprocessing, and using complementary instrumental techniques, the detection accuracy and specificity of adulteration can be increased, thus contributing to more effective food quality assurance.

## Figures and Tables

**Table 1:** Principal component regression (PCR) and partial least squares regression (PLSR) models, built with *f* latent variables for original and optimally preprocessed Fourier-transform near-infrared reflectance spectra of ground garlic with a selected adulterant (corn flour or corn starch).

Adulterant	Preprocessing	Model	*f*	RMSEC	RMSEP	R^2^c	R^2^v
Corn flour	none	PCR *	3	2.5397	2.3115	0.9919	0.9787
Corn starch	none	PCR	9	2.3140	2.6896	0.9933	0.9711
Corn starch	SNV	PCR *	4	2.2659	2.1256	0.9936	0.9820
Corn flour	none	PLSR	6	1.4365	1.5655	0.9974	0.9902
Corn flour	1st derivative ^1^	PLSR *	4	1.8841	1.8844	0.9955	0.9858
Corn starch	none	PLSR	8	1.7759	2.2297	0.9960	0.9802
Corn starch	ISC	PLSR *	4	1.7679	1.7812	0.9961	0.9873

^1^ The first-derivative spectra were obtained using Savitsky–Golay smoothing with a widow containing fifteen sampling points and a second-degree polynomial. * Asterisks indicate the optimal calibration models.

**Table 2:** Correct discrimination/classification rates (CCRs), sensitivities, and specificities reported as mean values obtained based on 500 training sets drawn randomly without replacement with the uncertainty of a given figure of merit expressed as the corresponding standard deviation for the optimal partial least squares discriminant model (PLS-DA) with seven latent variables. The model was built to discriminate samples adulterated with two different components (corn flour and corn starch).

Validation Parameter	Training Set	Internal Test Set	Validation Set
CCR	97.05 ± 1.38	94.51 ± 3.59	99.66 ± 0.87
Sensitivity	97.86 ± 1.3	94.15 ± 5.94	99.96 ± 3.57
Specificity	98.02 ± 1.46	94.88 ± 5.23	99.36 ± 1.67

**Table 3:** The optimal OC-PLS models with seven latent variables that were built to classify samples adulterated with corn flour or corn starch. Each model is characterized by sensitivity and specificity, and the correct classification rate (CCR) is calculated for training, test, and validation sets.

Model	Sensitivity	Specificity	CCR		
			Training	Test	Validation
Corn flour	97.33%	64.00%	96.00%	100%	64.00%
Corn starch	94.67%	45.33%	92.00%	100%	45.33%

## Data Availability

Data used in this study are available upon request from the corresponding author.
